# Characteristics of persons who inject drugs and who witness opioid overdoses in Vietnam: a cross-sectional analysis to inform future overdose prevention programs

**DOI:** 10.1186/s12954-017-0188-4

**Published:** 2017-09-07

**Authors:** N.A. Blackburn, K.E. Lancaster, T.V. Ha, C.A. Latkin, W.C. Miller, C. Frangakis, V.A. Chu, T. Sripaipan, V.M. Quan, N.L. Minh, P.T. Vu, V.F. Go

**Affiliations:** 1 Department of Health Behavior, Gillings School of Global Public Health, University of North Carolina at Chapel Hill, 302 Rosenau Hall, Chapel Hill, NC 27599 USA; 2 Division of Infectious Diseases, School of Medicine, University of North Carolina at Chapel Hill, 130 Mason Farm Road, Chapel Hill, NC 27599 USA; 3 University of North Carolina, No 6, Lane 76, Linh Lang Street, Hanoi, Vietnam; 4 Department of Health, Behavior, and Society, Johns Hopkins Bloomberg School of Public Health, 624 N. Broadway, Hampton House 737, Baltimore, Maryland 21205 USA; 5 Current affiliation: Division of Epidemiology, College of Public Health, The Ohio State University, 300-D Cunz Hall, 1841 Neil Avenue, Columbus, Ohio 43210 USA; 6 Department of Biostatistics, Johns Hopkins Bloomberg School of Public Health, 615 N. Wolfe Street, Room E3642, Baltimore, MD 21205 USA; 7 Department of Epidemiology, Johns Hopkins Bloomberg School of Public Health, 615 N. Wolfe Street, Baltimore, Maryland 21205 USA; 8 Centre for Preventive Medicine of Thai Nguyen, 971 Duong Tu Minh Road, Thai Nguyen, Vietnam

**Keywords:** Overdose, Persons who inject drugs (PWID), Vietnam, Overdose-reversal, Opioids

## Abstract

**Background:**

Persons who use opioids have a high risk of overdose and associated mortality. In Vietnam, little is known about the characteristics of this population and the persons who are witness to those overdoses. One approach to combatting fatal overdose has been the use of peer interventions in which a friend or injecting partner administers overdose reversal medication, but availability in Vietnam of these medications is limited to pilot programs with aims to expand in the future (Le Minh and V.F. Go, Personal Communication, 2016). The primary objective of this paper is to explore the characteristics associated with witnessing three or more overdoses in a lifetime.

**Methods:**

This cross-sectional analysis used baseline data from a four-arm randomized control trial conducted in Thai Nguyen, Vietnam, known as the Prevention for Positives project. One thousand six hundred seventy-three PWID were included in the analysis. We conducted bivariable and multivariable logistic regression to identify characteristics associated with witnessing three or more overdoses in a lifetime. Characteristics explored included education, employment, marital status, risky drug use behaviors, locations for accessing syringes, recent overdose, history of incarceration, drug treatment, and having slept outside in the past 3 months.

**Results:**

Seventy-two percent (*n* = 1203) of participants had witnessed at least one overdose in their lifetime, and 46% had witnessed three or more overdoses (*n* = 765). In the multivariable model, having less than secondary education (AOR 0.70; 95% CI 0.57, 0.86), having slept outside in the past 3 months (AOR 1.77; 95% CI 1.31, 2.40), having a history of incarceration (AOR 1.33; 95% CI 1.07, 1.65), having a history of drug treatment (AOR 1.41; 95% CI 1.12, 1.77), experiencing a recent non-fatal overdose (AOR 3.84; 95% CI 2.36, 6.25), injecting drugs daily (AOR 1.79; 95% CI 1.45, 2.20), receptive needle sharing (AOR 1.30; 95% CI 1.04, 1.63), and number of years injecting (AOR 1.04; 95% CI 1.02, 1.07) were significantly associated with witnessing three or more overdoses.

**Conclusions:**

Targeted interventions are needed to train persons witnessing an overdose to administer overdose-reversal medication. This includes targeting persons prior to release from prisons, drug treatment centers, and those accessing syringe exchange programs. Additional research should assess the burden of witnessing an overdose as well as locations for medication distribution. Assessments of the training capacity and needs for implementing these programs among drug using peers in Vietnam are of the utmost importance.

## Background

Overdoses from opioids are a global problem. The United Nations on Drugs and Crime (UNODC) has noted that 33 million people use opioids. When excluding people who abuse prescribed opioids, an issue largely isolated to North America, 17 million people in the world use opioids and approximately two thirds of those individuals live in Asia [1]. An opioid overdose can lead to death as well as other morbidities including brain damage [2].

Most of the world’s opium production occurs in southwest and southeast Asia. In much of Asia, the most commonly used opiate is heroin [1]. Asia has one of the highest crude mortality rates among opioid-using individuals of any region in the world with an estimated 5.23 deaths per 100 person-years [3]. Opioid overdose prevalence estimates are limited in large part because of the hard-to-reach drug using population. Many overdoses are never reported as people who witness an overdose often do not contact health authorities due to fear of arrest for drug-using behavior [4, 5].

The UNODC and the World Health Organization have recommended distribution of opioid-reversal drugs to persons who inject and their family and friends [2, 6]. Peer-based interventions are needed, as most opioid overdoses occur in the presence of other persons who inject drugs (PWID) [7, 8]. Opioid-reversal drugs, known as naloxone, prevent death effectively. Naloxone exists as both an injection and as a nasal spray, though in Vietnam only the injection version is currently available. Most often, people administer naloxone by injection; therefore, effective overdose prevention programs require a hands-on training [9]. A possible criticism of this approach is the concerns of non-medical personnel in using a syringe when administering the drug. However, if the people who use drugs are trained in recognizing overdose and administering naloxone, they are just as successful in reversing overdoses as medical professionals [7]. There are few contraindications for administering naloxone; persons who are administered the opioid receptor antagonist experience few side effects. Even when a person administers naloxone to someone they believe to be experiencing an opioid overdose but is not, the drug has a low likelihood of negative health effects [10]. In addition, naloxone distribution programs that target people who use opioids and family members of those who have a history of overdose from opioids are cost-effective [11].

The World Health Organization recommends providing naloxone to those who are likely to witness an overdose [2, 12]. However, naloxone distribution and other overdose prevention interventions have historically focused on medical personnel and only recently have begun to implement programs with drug using peers and/or family members [13]. In Vietnam, naloxone availability currently is limited to a few pilot programs in Hanoi and Ho Chi Minh City, with plans to expand naloxone programs in the near future [14]. To inform scale up efforts throughout Vietnam, we sought to define the characteristics of potential administrators of naloxone: PWID who have witnessed three or more overdoses. In doing so, we aim to identify populations to focus on for implementation of overdose prevention interventions in Vietnam.

## Methods

### Study location

Participants were recruited in Thai Nguyen, a northeastern province of Vietnam approximately 80 km north of the capital city Hanoi near the border with China. Recent estimates suggest about 6000 PWID live in this part of Vietnam [15]. The most frequently used drug in this area is heroin. Heroin is inexpensive in Vietnam and readily available in this part of the world because of the geographic location of the trade route from the Golden Triangle [4].

### Study design and study population characteristics

This cross-sectional analysis used baseline data collected as part of a four-arm randomized control trial known as the Prevention for Positives project. The parent trial evaluated the effectiveness of a multi-level intervention to reduce high-risk injecting and sexual practices among HIV-positive PWID in order to reduce forward HIV transmission. Details of this trial are described elsewhere [16], but briefly, participants were recruited through peer referral in which a team of seven current and former drug users approached their drug networks with information about the trial and referred them for screening. Inclusion criteria for the trial included (1) being male, (2) being 18 years old, (3) being sexually active in the past 6 months, (4) having self-reported injection drug use in the past 6 months, and (5) being able to provide informed consent. A total of 1739 PWID were screened and 1674 deemed eligible and consented for participation in the baseline interview. Trained study staff administered a 1-h face-to-face interview using a questionnaire that included questions on demographic characteristics, sexual risk behaviors, drug use behaviors including equipment sharing, and stigma regarding areas such as HIV and injection drug use. Not all participants who completed the questionnaire completed the full trial but are included in this cross-sectional analysis. All participants received 75,000 Vietnamese Dong ($3.50 USD) as well as reimbursement for the cost of travel to the study site. This research protocol was approved by the Thai Nguyen Center for Preventive Medicine Institutional Review Board and Johns Hopkins Institutional Review Board.

### Measures

The dependent variable for this analysis is having ever witnessed three or more overdoses. Prior to answering questions about history of witnessing an overdose, interviewers provided a definition of overdose: “Drug overdose happens when an individual takes more drugs than the body can handle. When an individual overdoses on a drug, he may have some of the following symptoms: throwing-up, face turning pale, unable to talk, slow and erratic heartbeats, slow and shallow breathing, losing consciousness, etc.” After assessing the frequency and distribution of overdoses, we selected witnessing three or more overdoses as we found that among those witnessing at least one, almost half (46%) had witnessed three or more; other studies have noted that these people who report a history of being witness to more than one overdose are more likely to witness a future overdose [17, 18]. We first defined this variable using the survey question, “In your lifetime, how many people have you witnessed having an overdose (not including yourself)?” Those who responded with three or more were coded as “yes” to having witnessed three or more overdoses in their lifetimes and those who responded with zero, one, or two were coded as “no” for having witnessed three or more overdoses in their lifetimes.

We identified the primary independent variables for this analysis from the overdose literature and from our own hypotheses based on our conceptualization of witnessing an overdose. We assessed recent experience of an overdose (How many different times in the past 12 months have you overdosed?). Those who responded with one or more were coded as “yes” to having experienced a recent overdose and those who responded with zero were coded as “no” for having experienced a recent overdose [17]. Important for populations of PWID, we determined additional key independent variables for analysis including history of incarceration (Have you ever been incarcerated (that is being put in prison or jail or detention center)?) [18] as well as having slept outside in the past 3 months based on literature around homelessness and housing instability in drug-using populations [19] (During the last 3 months have you ever spent a night on the street, in a park, in an alley, or in an abandoned building?).

We hypothesized that high-risk drug use behaviors such as daily injecting of drugs and using a needle after someone else (receptive needle sharing) would be associated with having been a witness to an overdose [20]. We included a history of drug treatment (Have you ever been in a drug treatment program?) because drug treatment can often be mandated as part of legal punishment of illicit drug use [16].

We also captured data on location of needles purchased in the past 3 months, including pharmacies which are the dominant location for inexpensive clean needle purchases in Thai Nguyen [16]. We hypothesized that individuals who did not obtain their needles from a pharmacy may be engaging in riskier drug use behaviors and therefore more likely to have witnessed three or more overdoses. Additionally, we included the number of years a person reported injecting drugs since those who have injected drugs longer may have an increased likelihood for being a witness to an overdose [18]. Other demographic variables included education (less than a high school education versus more than a high school education), marital status (married or living with partner versus not married or living with partner), and employment status (full-time employment being 30 h a week or more).

### Statistical analyses

The full sample of participants was 1674. We removed one person from the analysis because of their response to the primary outcome, having ever witnessed an overdose, being “Don’t Know,” and thus had a remaining sample of 1673. We calculated frequency distributions for categorical variables and medians and inter-quartile ranges for continuous variables. We conducted bivariable logistic regression to estimate unadjusted odds ratios and 95% confidence intervals and for characteristics associated with those having witnessed three or more overdoses in their lifetimes; we determined that the high proportion of the population having been witness to three or more overdoses warranted further exploration.

To estimate adjusted odds ratios and 95% confidence intervals, we utilized simultaneous entry for all variables associated with witnessing three or more overdoses in bivariable analyses, using a *p* value of < 0.10. We also included characteristics previously identified in the literature as significant among this population having witnessed three or more overdoses. Additionally, we controlled for age in the multivariable analysis because witnessing an overdose, both fatal and non-fatal, has been associated with increasing age [21, 22]. We adjusted for drug treatment in this final adjusted model recognizing that the directionality of the relationship of history of drug treatment has been more difficult to tease apart since it has been hypothesized that individuals may seek treatment after witnessing an overdose [17]. For independent variables with a response of “Don’t Know,” we coded these as missing and did not include them in the analysis. Characteristics with a *p* value of < 0.05 in the final adjusted model were considered independently associated with having witnessed three or more overdoses. We used Pearson’s correlation coefficient to assess collinearity of all independent and dependent variables prior to testing the full adjusted model.

## Results

### Participant characteristics

We sampled 1673 participants. The median age was 36 (IQR 28–44), and half reported being married or living with a partner (Table 1). Most reported full-time employment (73.9%). More than half reported injecting drugs on a daily basis (51.3%), and most reported purchasing their needles from a pharmacy (86.7%). About one third of the sample reported having been in a drug treatment program (30.7%), and similarly, more than one third of the sample reported having ever been incarcerated (35.0%).

**Table 1:** Characteristics of persons who inject drugs (PWID) living in Thai Nguyen, Vietnam, in a multi-arm randomized control trial (*n* = 1673)

	Total study population (*N* = 1673)	Witness to 3 or more overdoses (*N* = 765)	Witness to less than 3 overdoses (*N* = 908)
Baseline characteristics	*n* (%)	*n* (%)	*n* (%)
Age, median (IQR) (years)	36 (28–44)	35 (28–42)	36 (28–44)
Education			
High school or more	702 (42.0)	362 (47.3)	340 (37.4)
No high school	971 (58.0)	403 (52.7)	568 (62.6)
Marital status			
Not married or living with a partner	853 (51.0)	403 (52.7)	450 (49.6)
Married or living with a partner	820 (49.0)	362 (47.3)	458 (50.4)
Employment status			
Less than full-time	436 (26.1)	223 (29.1)	213 (23.5)
Full-time	1237 (73.9)	542 (70.9)	695 (76.5)
Spent the night outside in the past 3 months^a^			
No	1428 (85.4)	613 (80.1)	815 (89.8)
Yes	245 (14.6)	152 (19.9)	93 (10.2)
Lifetime history of incarceration			
No	1087 (65.0)	450 (58.9)	637 (70.1)
Yes	585 (35.0)	314 (41.1)^c^	271 (29.9)
Lifetime history of drug treatment			
No	1160 (69.3)	481 (62.9)	679 (74.8)
Yes	513 (30.7)	284 (37.1)	229 (25.2)
Recent non-fatal overdose^b^			
No	1569 (93.8)	685 (89.5)	884 (97.4)
Yes	104 (6.2)	80 (10.5)	24 (2.6)
Daily injecting of drugs			
No	815 (48.7)	298 (38.9)	517 (56.9)
Yes	858 (51.3)	467 (61.1)	391 (43.1)
Needle purchasing behavior purchased at a pharmacy^a^			
No	222 (13.3)	106 (13.9)	116 (12.8)
Yes	1450 (86.7)	659 (86.1)	791 (87.1)
Don’t Know	1 (0.1)	0 (0.0)	1 (0.1)
Receptive needle sharing behavior			
No	1120 (66.9)	478 (62.5)	642 (70.7)
Yes	553 (33.1)	287 (37.5)	266 (29.3)
Lifetime number of years injecting drugs, median (IQR) (years)^d^	8 (4–12)	9 (5–13)	7 (3–11)

*IQR* interquartile range

^a^Past 3 months

^b^Past 12 months

^c^One individual missing information about incarceration history

^d^Thirteen responses “Don’t Know” (*n* = 1613)

### Associations with having witnessed three or more overdoses

**Fig. 1:**
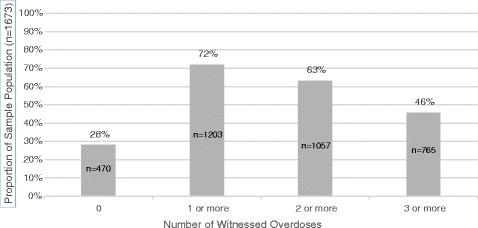
Number of participants who report witnessing an overdose in Thai Nguyen, Vietnam (*n* = 1673)

More than two thirds of the total sample reported witnessing at least one overdose (71.9%), 1057 persons witnessed two or more overdoses (63.2%), and 765 persons reported witnessing three or more overdoses in their lifetimes (45.7%) (Fig. 1). Of the 765 people who reported witnessing three or more overdoses, 47.3% were married and living with a partner and 58.0% had less than a high school education.

In bivariable analyses, those who witnessed three or more overdoses had higher odds of experiencing a recent overdose themselves (OR 4.30; 95% CI 2.70, 6.86), of reporting having slept outside in the past 3 months (OR 2.17; 95% CI 1.65, 2.87), of having ever been incarcerated (OR 1.64; 95% CI 1.34, 2.01), of having ever been in drug treatment (OR 1.75, 95% CI 1.42, 2.16), of injecting drugs daily (OR 2.07; 95% CI 1.70, 2.52), of engaging in receptive needle sharing behavior (OR 1.45; 95% CI 1.18, 1.78), and of having a longer injecting history (OR 1.07; 95% CI 1.04–1.09) (Table 2). Those who had witnessed three or more overdoses had lower odds of being employed full-time (OR 0.75; 95% CI 0.60–0.93) and having less than a high school education (OR 0.66; 95% CI 0.55, 0.81) than those who had witnessed fewer than three overdoses.

**Table 2:** Bivariable and multivariable associations with having witnessed three or more overdoses among individuals injecting drugs in Thai Nguyen, Vietnam, *n* = 1673

Characteristic	OR	(95% CI)	*p* value	AOR^a^	(95% CI)	*p* value
Education						
High school or more	1.00			1.00		
No high school	0.66	(0.55–0.81)	< 0.01	0.70	(0.57–0.86)	< 0.01
Marital status						
Not married or living with a partner	1.00					
Married or living with partner	0.88	(0.73–1.07)	0.20			
Employment Status						
Less than full-time	1.00			1.00		
Full-time	0.75	(0.60–0.93)	0.01	0.84	(0.66–1.06)	0.1313
Spent the night outside in the past 3 months						
Did not spend the night outside	1.00			1.00		
Spent the night outside	2.17	(1.65–2.87)	< 0.01	1.77	(1.31–2.40)	< 0.01
Lifetime history of incarceration^b^						
No history of incarceration	1.00			1.00		
History of incarceration	1.64	(1.34–2.01)	< 0.01	1.33	(1.07–1.65)	0.101
Lifetime history of drug treatment						
No history of drug treatment	1.00			1.00		
History of drug treatment	1.75	(1.42–2.16)	< 0.01	1.41	(1.12–1.77)	< 0.01
Non-fatal overdose						
No recent non-fatal overdose	1.00			1.00		
Recent non-fatal overdose	4.30	(2.70–6.86)	< 0.01	3.84	(2.36–6.25)	< 0.01
Drug injecting frequency						
No daily injecting of drugs	1.00			1.00		
Daily injecting of drugs	2.07	(1.70–2.52)	< 0.01	1.79	(1.45–2.20)	< 0.01
Needling purchasing behavior						
Did not purchase at a pharmacy	1.00					
Purchased at a pharmacy^c^	0.91	(0.69–1.21)	0.52			
Receptive needle sharing behavior						
Did not share needles	1.00			1.00		
Shared needles	1.45	(1.18–1.78)	< 0.01	1.30	(1.04–1.63)	0.02
Lifetime number of years injecting						
Years of injecting	1.07	(1.04–1.09)	< 0.01	1.04	(1.02–1.07)	< 0.01

*OR* odds ratio, *AOR* adjusted odds ratio

^a^Adjusted for age and those variables with *p* value < 0.10 in bivariable analyses were included in the final multivariable model: education, employment status, slept outside in the past 3 months, history of incarceration, history of drug treatment, recent non-fatal overdose, daily injecting of drugs, and receptive needle sharing behavior; *n* = 1667

^b^One response missing (*n* = 1672)

^c^One response “Don’t Know” (*n* = 1672)

In multivariable analysis (Table 2), variables that remained significant in the adjusted model after controlling for age included having slept outside in the past 3 months (AOR 1.77; 95% CI 1.31, 2.40), history of drug treatment (AOR 1.41; 95% CI 1.12, 1.77), receptive needle sharing behavior (AOR 1.30, 95% CI 1.04, 1.63), and years injecting (AOR 1.04; 95% CI 1.02, 1.07). History of incarceration was also significant in the full model (AOR 1.33; 95% CI 1.07, 1.65). Individuals who had less than high school education (AOR 0.77; 95% CI 0.62, 0.96) were less likely to witness three or more overdoses. Employment was not significant in the full model.

## Discussion

Almost half of our sample had witnessed three or more drug overdoses and more than 70% had witnessed at least one, mirroring worldwide estimates [23]. Those who reported receptive needle sharing were more likely to witness three or more overdoses, and more than half of the sample witnessing three or more overdoses reported injecting drugs on a daily basis. These characteristics suggest a population engaging in high-risk drug use behavior and doing so with other PWID. We found people who had recently overdosed themselves were more likely to witness three or more overdoses. Overdose has historically been explored as an individual experience and problem, but recent recommendations from WHO and UNODC to distribute naloxone to the family and friends of PWID [24] suggest a changing approach. These recommendations to address the more social aspect of overdose must be coupled with research to better understand the social context in which individuals inject drugs. PWID who overdose are also more likely to be with another partner or group when the event occurs [25]. Most overdose deaths do not occur instantaneously, suggesting there is a window of time in which peers witness to the overdose, can recognize symptoms, and administer naloxone [26].

In addition to high-risk drug use behaviors, our analyses found that those persons who reported sleeping on the street in the past 3 months were more likely to witness three or more overdoses. In a 2015 study among younger PWID in Hanoi, Vietnam, nearly 10% reported living on the street with another 19% reporting temporary housing [25]. Persons who suffer a fatal overdose are more likely to suffer from instability in day to day life, such as homelessness [27]. Housing instability may also impact their ability to keep a job or maintain intimate relationships. Such characteristics highlight the challenges in targeting this population since they may be difficult to reach for interventions [28, 29]. Our measure asked about sleeping outside in the past 30 days; thus, measures that can better identify the type of housing instability and possible homelessness are needed in order to identify venues for potential recruitment of people likely to witness three or more overdoses including centers that provide housing and services to homeless individuals. Our findings also suggested that drug treatment centers and prisons may be possible venues for reaching individuals likely to witness three or more overdoses.

In Vietnam, some non-profit organizations have begun to distribute naloxone to non-medical professionals and the general population as part of pilot programs [14]. Needle and syringe exchange programs (NSPs) are also possible locations for distributing naloxone to those who are injecting drugs daily [30]. During the time of data collection, there were 16 needle exchange sites operating in Thai Nguyen [16], thus a possible avenue for distribution in the province. The majority of participants in our sample reported using pharmacies for accessing syringes, which we attribute to the fact that pharmacies sell clean needles inexpensively and are readily available throughout the province. An option that has been explored in parts of North America is to distribute naloxone through pharmacies [13]. In the USA, programs using pharmacists as the source of opioid overdose prevention programs are expanding, using the healthcare system as the avenue for distribution of naloxone [13, 30]. However, our analyses did not find a significant relationship between buying needles from a pharmacy and being a witness to three or more overdoses. Indeed, we found that those who bought needles at pharmacies engaged in lower risk drug use behavior compared to those who did not access pharmacies. Other research suggest that pharmacies are a good avenue for syringe distribution to PWID [31]; therefore, pharmacies may be a good area for research in overdose prevention in Vietnam.

As distribution of overdose-reversal drugs are expanded in Vietnam, interventions to train drug users on how to recognize an overdose, how to distribute naloxone, and how to administer it will need to be developed [9]. In a recent study in Hanoi, Vietnam, knowledge about overdose, including how to identify someone experiencing an overdose and what steps to take to prevent death from an overdose, varied greatly [4]. The development and implementation of these trainings in Vietnam would support recent recommendations from the World Health Organization that countries should expand access to naloxone so that laypersons could acquire it [2]. In the USA, researchers have developed the Opioid Overdose Knowledge scale which supports the training of family members and peers of drug users to administer naloxone [32]. Adaptation of this scale for the Vietnamese setting may support the effectiveness of the trainings in naloxone. Research and further training on overdose prevention should also incorporate where the overdoses are most frequently occurring. US-based studies have found that overdoses are frequently witnessed at home or in private locations [33, 34]. By identifying such areas in the Vietnamese context, education and training could be further tailored to the population most at risk.

It is important to recognize our limitations from these analyses. This is a sample of individuals who were residents of Thai Nguyen, male, reported having injected drugs, and interested in enrolling into an HIV- and injection drug use (IDU)-related stigma reduction intervention study. Individuals referred their peers to the study which limits our ability to generalize to the larger drug-using population in Vietnam. Other studies conducted in this area of Vietnam have found that 97% of PWID are male [4]. Self-report of witnessing an overdose is an additional limitation of these data as it may have introduced social desirability bias as well as recall bias; thus, the number of individuals who have been a witness to an overdose may be higher than is presented here. These data are cross-sectional, and therefore, we are unable to determine temporal relationships with the characteristics associated for having witnessed an overdose. Specifically, the directionality of the relationship of history of drug treatment has been more difficult to tease apart since it has been hypothesized that individuals may seek treatment after witnessing an overdose [17]. Additionally, participants only provided detail on drug treatment if it was in the home or outside of the home. Information on types of drug treatment received, including methadone, were not collected.

## Conclusions

In Vietnam, individuals who are a witness to three or more overdoses may have unique characteristics beyond injecting drugs that public health programs should target for intervention including having experienced a recent non-fatal overdose, housing instability, and incarceration. Collaboration with prisons and drug treatment centers to conduct trainings on overdose prevention pre-release is a potential starting point for interventions. Furthermore, identification of gathering places of those recently released from prison or those without housing may help public health officials in Vietnam to identify populations ideal for opiate overdose prevention interventions among persons who use drugs.

## Abbreviations

IDU: Injection drug use
NSPs: Needle and syringe exchange programs
PWID: Persons who inject drugs
UNODC: United Nations Office on Drugs and Crime
WHO: World Health Organization
